# Low Awareness of Overweight Status Among Parents of Preschool-Aged Children, Minnesota, 2004-2005

**Published:** 2009-03-15

**Authors:** Lisa Harnack, Leslie Lytle, John H. Himes, Mary Story, Gretchen Taylor, Don Bishop

**Affiliations:** Division of Epidemiology and Community Health, University of Minnesota; University of Minnesota, Minneapolis, Minnesota; University of Minnesota, Minneapolis, Minnesota; University of Minnesota, Minneapolis, Minnesota; Minnesota Department of Health, Minneapolis, Minnesota; Minnesota Department of Health, Minneapolis, Minnesota

## Abstract

**Introduction:**

Many studies have found that parents of overweight children do not perceive their child to be overweight. Little is known, however, about the extent to which such misperceptions exist among parents of preschool-aged children.

**Methods:**

We analyzed data that were collected in 2004-2005 from parents of 593 preschool-aged children in 20 child care centers in the Minneapolis-St. Paul, Minnesota, metropolitan area. Parents were asked how they would classify their preschooler's weight, and children's height and weight were measured.

**Results:**

Of the predominantly white, educated sample, most parents (90.7%) of overweight preschoolers classified their child as normal weight. An even higher percentage (94.7%) of children at risk for overweight were classified as normal weight by their parents. Most parents of normal-weight children classified their child's weight as average. However, 16.0% classified their normal-weight child as underweight or very underweight.

**Conclusion:**

Results indicate that parents are unlikely to recognize childhood overweight among preschool-aged children, which is concerning because parents of overweight children may be unlikely to engage in obesity prevention efforts for their child if they do not recognize their child's risk status. A notable proportion of parents of normal-weight children perceived their child to be underweight, which suggests that parents of normal-weight children may be more concerned with undernutrition than overnutrition.

## Introduction

Over the past several decades the prevalence of childhood overweight has been increasing in the United States (1,2). The prevalence rate is alarming, especially among preschool-aged children. National survey data indicate that 13.9% of children aged 2 to 5 years are overweight and 12.3% are at risk for overweight (2). Childhood overweight is associated with adverse health outcomes, and childhood body mass index (BMI) influences adult adiposity (3). Furthermore, a recent study found that children who were at or above the 85th BMI percentile during the preschool period were more than 5 times more likely to be overweight at age 12 than those at a low BMI percentile (4).

Parents of overweight children often do not perceive their child to be overweight (5-15). This problem is particularly acute among younger children (6,8,14,15). Low recognition of childhood overweight may be problematic because parents may be less likely to prevent their children from becoming obese if they do not recognize their children's risk status.

Few studies have evaluated parents' perceptions of their preschool-aged children's weight (8,14). Parental perceptions may be more accurate in this age group because annual well-child pediatric visits are recommended between the ages of 2 to 5 years (16). Height and weight are routinely measured at these visits to track child growth, which presents the opportunity for determination of weight status by the health care provider. Conversely, parental perceptions may be poor in this age group because cultural perceptions of weight in early childhood differ (8,17). To contribute to understanding parental weight perceptions in preschool-aged children, we analyzed data collected in 2004-2005 from parents of 593 children enrolled in child care centers in the Minneapolis-St. Paul, Minnesota, metropolitan area.

## Methods

Data were collected as part of 5 A Day Preschool Power Plus, a group-randomized trial to evaluate a child care center–based program to increase fruit and vegetable intake of preschool-aged children. Twenty child care centers participated in the trial. The centers, all owned by the same company, were located in both urban and suburban communities. Study activities (measurements and implementation of the program) were conducted in 2 waves; 10 centers participated in the first wave (2004-2005 school year), and 10 centers participated in the second wave (2005-2006 school year).

Measures collected before centers were randomized to intervention or control groups (baseline measures) included a self-administered parent questionnaire that was distributed to parents of children in the preschool-age classrooms in each center. A parent's or guardian's perception of their child's weight status was assessed with the question, "How would you classify your preschooler's weight?" Response options were "very underweight," "underweight," "average weight," "overweight," and "very overweight." Demographic information such as parents' race and education level was also collected in the parent questionnaire, and parents were asked to self-report their height and weight. Children's height and weight were measured at the child care centers by trained study staff following standardized procedures (18). Height was measured to the nearest 0.1 cm using a portable stadiometer (Invicta, London, United Kingdom), and weight was measured to the nearest pound on a digital scale (seca gmbh & co, Hamburg, Germany). Children were wearing light clothing and no shoes when their measurements were taken.

A total of 706 children were eligible for participation in the study at baseline. Measures of height and weight were collected from 676 (95.8%) of these children, and 621 (88.0%) parent surveys were collected. Analyses were restricted to children for whom both height and weight measures were collected and a parent survey was completed, resulting in a sample of 593 (84.0% of eligible children).

BMI (kg/m^2^) was calculated for each child, and BMI percentiles were determined using the 2000 Centers for Disease Control and Prevention BMI-for-age growth charts for children aged 2 to 20 years (19). Using these percentiles, children were classified as underweight (≤5th percentile), normal weight (6th-84th percentile), at risk for overweight (85th-94th percentile), or overweight (≥95th percentile).

Descriptive statistics (means and frequencies) were computed. Logistic regression analyses were conducted to compare characteristics of parents who incorrectly classified their child’s weight status with those who correctly classified their child’s weight status. For these analyses the following classifications were considered correct: 1) very underweight and underweight categories are correct classifications for a BMI less than or equal to the 5th percentile, 2) average weight category is the correct classification for a BMI from the 6th to the 84th percentile, and 3) overweight or very overweight categories are the correct classifications for a BMI greater than or equal to the 85th percentile. All analyses were conducted using SAS (SAS Institute, Cary, North Carolina).

Informed consent was collected from parents following procedures approved by the institutional review boards of the Minnesota Department of Health and University of Minnesota.

## Results

Children ranged in age from 2 to 5 years; most were aged 3 to 4 years (Table 1). The sample was predominantly white (86.5%), and most of the parent surveys were completed by a woman (90.5%). Approximately 90% of the parents who completed the survey had more than a high-school education, and 60% had a college degree.

Most of the children (80.4%) had a BMI greater than the 50th percentile (Table 1). About one-fourth (25.5%) of the children were at risk for overweight (BMI 85th-94th percentile), and 12.6% were overweight (BMI ≥95th percentile). Sixty-one percent were normal weight (BMI 6th-84th percentile). Three children (0.5%) were underweight (BMI for age ≤5th percentile).

Most parents (87.4%) classified their child as average weight. A small number of parents classified their child as overweight (1.4%; n = 8) or very overweight (0.3%; n = 2). In contrast, 63 parents (10.6%) classified their child as underweight and 2 (0.3%) classified their child as very underweight (data not shown).

Most (90.7%) parents of overweight preschoolers classified their child as average weight, 6.7% classified their child as overweight, and 2.7% classified their child as very overweight (Table 2). Most children at risk for overweight (94.7%) were classified by their parents as average weight, and 4.0% were classified as underweight. Most parents of normal-weight children correctly classified their child's weight as average (83.8%). However, 16.0% incorrectly classified their normal-weight child as underweight or very underweight. One parent classified a normal-weight child as overweight.

Overall, 278 parents (46.9%) misclassified their child's weight status. More (275, 46.4%) children were classified as belonging to a lower weight category than was actually the case, and only 3 children (0.5%)  were placed into a higher category (Table 2). Older parents were less likely to incorrectly classify their child's weight status compared with younger parents (adjusted odds ratio, 0.66; 95% confidence interval, 0.44-1.00), and this difference approached significance (Table 3).

## Discussion

Our findings indicate that parental recognition of childhood overweight among preschool-aged children is poor. This finding is concerning because parents of overweight children or children at risk for overweight may be less likely to actively engage in obesity prevention efforts for them if they do not recognize their child's risk status. A notable proportion of parents of normal-weight children perceived their child to be underweight. This finding suggests that parents of normal-weight children may be more concerned with undernutrition than overnutrition. Childhood obesity prevention messages are unlikely to be heeded by some parents of normal-weight children if this is the case.

In contrast to previous studies that focused on low-income and ethnically diverse samples of parents (8,14), parents in our study were predominantly white and well educated. Nonetheless, our findings are consistent with earlier reports in preschool-aged children (8,14), suggesting that poor parental recognition of childhood overweight is a widespread problem. A 1998-1999 survey of low-income mothers of children aged 23 to 60 months found that only 29% of mothers of overweight children believed their child was overweight. Similarly, in a 2003 survey of WIC (Special Supplemental Program for Women, Infants and Children) participants in New York City, 93% of parents of overweight children aged 2 to 4 years reported that their child was just the right size or underweight (14).

Weight misperceptions may be high among parents of preschool-aged children who are overweight or at risk for overweight because of differences in cultural perceptions of weight in early childhood. Results from focus groups conducted with low-income mothers with children aged 12 to 36 months suggest that mothers believe that high infant weight is a sign of child health and good parenting (8). Indeed, none of the parents in the focus groups indicated that an infant could be too heavy nor could they identify any specific age at which an infant or toddler could be considered overweight. Similarly, in an ethnographic study of Latino families in New York City, mothers believed heavy children were "safer" and "less fragile" compared with thin children (17). Furthermore, overweight children tended to be viewed as normal weight in the context of families in which multiple generations of family members are overweight or obese.

Health care providers' lack of attention to weight issues in early childhood may be contributing to parental misperceptions of children's weight status. Although recommendations encouraging pediatricians to incorporate overweight assessment into routine clinical practice have been issued (20,21), results from several studies suggest this is not yet regularly occurring (22-24). Consequently, some parents of children who are overweight or at risk for overweight may assume their child is at a normal weight because the pediatrician has not discussed their child's weight with them.

Our study has a number of methodological strengths, including a high participation rate and height and weight measurements collected using appropriate equipment and trained staff. However, our sample was drawn from children enrolled in child care centers in 1 metropolitan area. This sampling yielded a predominantly white, educated sample. Strictly interpreted, study findings should be generalized to only similar populations.

Efforts are needed to educate parents of young children about their child's weight status. Although various avenues exist for intervening to improve parental awareness of child weight status, from a public health perspective the route that is most likely to reach the greatest proportion of parents efficiently is primary care. Annual well child pediatric visits are recommended between the ages of 2 to 5 years (16). Height and weight are routinely measured at these visits to track child growth, presenting the opportunity for determining weight status. Weight status results should be communicated to all parents, given that misperceptions exist among not only parents of overweight children but also parents of normal-weight children. Cultural perceptions that a high body weight in young children is healthy should be considered when communicating this information. For example, using terms such as "healthy weight" and "unhealthy weight" in place of terms such as "normal weight" and "overweight" may be helpful.

A key issue is equipping providers with the skills and resources to address the needs of children identified as overweight or at risk for overweight. Although research suggests that parents of overweight children are more apt to enter the preparation or action stage of change if they perceive their child's weight to be a health problem (25), whether effective and safe diet and physical activity changes will be instigated by parents if support services or resources are not provided in conjunction with providing body weight status information is unclear. Therefore, consideration must be given to this issue.

## Figures and Tables

**Table 1:** Baseline Characteristics of Survey Participants and Their Preschool-Aged Children, 5 A Day Preschool Power Plus Program, Minneapolis, Minnesota, 2004-2005

**Characteristic**	n (%)
**Sex of child (n = 593)**	
Female	274 (46.2)
Male	319 (53.8)
**Age of child, y (n = 593)**	
2	42 (7.1)
3	221 (37.3)
4	284 (47.9)
5	46 (7.8)
**BMI-for-age percentile of child^a^ (n = 593)**	
≤5th	3 (0.5)
6th-15th	16 (2.7)
16th-50th	97 (16.4)
51st-84th	251 (42.3)
85th-94th	151 (25.5)
≥95th	75 (12.6)
**Race/ethnicity of parent (n = 591)**	
Non-Hispanic white	511 (86.5)
White, Hispanic or Latino	19 (3.2)
Black/African American	21 (3.6)
Asian	22 (3.7)
Other	18 (3.0)
**Sex of parent (n = 589)**	
Female	533 (90.5)
Male	56 (9.5)
**Age of parent, y (n = 582)**	
18-25	56 (9.6)
26-35	279 (47.9)
36-55	247 (42.4)
**Highest level of parent education (n = 588)**	
Some high school	5 (0.9)
High school graduate or GED	52 (8.8)
Technical school or some college	173 (29.4)
≥College graduate	358 (60.9)

Abbreviations: BMI, body mass index; GED, general educational diploma.

a Based on 2000 Centers for Disease Control and Prevention's BMI-for-age growth charts for children aged 2 to 20 years. Underweight ≤5th percentile; normal weight 6th to 84th percentile; at risk for overweight 85th to 94th percentile; overweight ≥95th percentile.

**Table 2:** Parents' Perception of Preschool-Aged Child's Weight Status, by Measured Weight Status (n = 593), 5 A Day Preschool Power Plus Program, Minneapolis, Minnesota, 2004-2005

Parent Perception of Child's Weight Status	Weight Status of Children^a^			

	Underweight, No. (%) (n = 3)	Normal Weight, No. (%) (n = 364)	At Risk for Overweight, No. (%) (n = 151)	Overweight, No. (%) (n = 75)
Very underweight	0 (0)	2 (0.5)	0 (0)	0 (0)
Underweight	1 (33.3)	56 (15.4)	6 (4.0)	0 (0)
Average weight	2 (66.7)	305 (83.8)	143 (94.7)	68 (90.7)
Overweight	0 (0)	1 (0.3)	2 (1.3)	5 (6.7)
Very overweight	0 (0)	0 (0)	0 (0)	2 (2.7)

a Status determined by BMI-for-age percentiles of children aged 2 to 20 years, based on Centers for Disease Control and Preventions's growth charts. Underweight ≤5th percentile; normal weight 6th to 84th percentile; at risk for overweight 85th to 94th percentile; overweight ≥95th percentile.

**Table 3:** Factors Associated With Parent Misclassification of Child Weight Status^a^ (n = 565),^b^ 5 A Day Preschool Power Plus Program, Minneapolis, Minnesota, 2004-2005

**Characteristic**	AOR (95% CI)^c^
**Sex of child**	
Male	1 [Reference]
Female	1.11 (0.79-1.57)
**Age of child, y**	
2-3	1 [Reference]
4-5	0.95 (0.68-1.33)
**Race/ethnicity of parent**	
Non-Hispanic white	1 [Reference]
Other^d^	0.68 (0.41-1.12)
**Age of parent, y**	
18-30	1 [Reference]
31-55	0.66 (0.44-1.00)
**Education level of parent**	
≥College graduate	1 [Reference]
<College graduate	0.97 (0.66-1.42)
**BMI of parent,^e^ kg/m^2^**	
<25.0	1 [Reference]
≥25.0	1.34 (0.95-1.90)

Abbreviations: AOR, adjusted odds ratio; CI, confidence interval; BMI, body mass index.

a Parent's correct perception of weight status as "underweight or very underweight" (≤5th percentile), "average weight" (6th to 84th percentile), or "overweight or very overweight" (≥85th percentile) validated by BMI-for-age percentiles of children aged 2 to 20 years, based on Centers for Disease Control and Prevention growth charts.

b Number is less than the total sample size because children with missing information for 1 or more of the variables included in the table were excluded from the analysis.

c Odds ratios are adjusted for other variables included in the table.

d Includes participants who reported that they were “white, Hispanic or Latina/Latino,” “Black/African American,” “Asian,” “American Indian/Alaska Native,” “Native Hawaiian/other Pacific Islander,” or “other.”

e BMI <25.0 kg/m^2^ is normal weight, and BMI ≥25.0 kg/m^2^ is overweight.
